# Medical students review of formative OSCE scores, checklists, and videos improves with student-faculty debriefing meetings

**DOI:** 10.1080/10872981.2017.1324718

**Published:** 2017-05-19

**Authors:** Aaron W. Bernard, Gabbriel Ceccolini, Richard Feinn, Jennifer Rockfeld, Ilene Rosenberg, Listy Thomas, Todd Cassese

**Affiliations:** ^a^ Frank H. Netter MD School of Medicine at Quinnipiac, Hamden, CT, USA

**Keywords:** OSCE, feedback, clinical skills, self-assessment, video review SP: Standardized patient

## Abstract

**Background:** Performance feedback is considered essential to clinical skills development. Formative objective structured clinical exams (F-OSCEs) often include immediate feedback by standardized patients. Students can also be provided access to performance metrics including scores, checklists, and video recordings after the F-OSCE to supplement this feedback. How often students choose to review this data and how review impacts future performance has not been documented.

**Objective:** We suspect student review of F-OSCE performance data is variable. We hypothesize that students who review this data have better performance on subsequent F-OSCEs compared to those who do not. We also suspect that frequency of data review can be improved with faculty involvement in the form of student-faculty debriefing meetings.

**Design:** Simulation recording software tracks and time stamps student review of performance data. We investigated a cohort of first- and second-year medical students from the 2015-16 academic year. Basic descriptive statistics were used to characterize frequency of data review and a linear mixed-model analysis was used to determine relationships between data review and future F-OSCE performance.

**Results:** Students reviewed scores (64%), checklists (42%), and videos (28%) in decreasing frequency. Frequency of review of all metric and modalities improved when student-faculty debriefing meetings were conducted (p<.001). Among 92 first-year students, checklist review was associated with an improved performance on subsequent F-OSCEs (p = 0.038) by 1.07 percentage points on a scale of 0-100. Among 86 second year students, no review modality was associated with improved performance on subsequent F-OSCEs.

**Conclusion:** Medical students review F-OSCE checklists and video recordings less than 50% of the time when not prompted. Student-faculty debriefing meetings increased student data reviews. First-year student’s review of checklists on F-OSCEs was associated with increases in performance on subsequent F-OSCEs, however this outcome was not observed among second-year students.

## Introduction

Clinical skills development requires time and practice. Feedback has been shown to speed up the rate of skill development and promote higher levels of expertise [1,2]. An educator therefore should provide learners with specific, timely, and actionable feedback [3,4]. To optimize experiential learning and improve future performance, a learner should enter a period of reflection during which available feedback is examined [5]. A goal of medical education is to teach students to become self-directed, life-long learners, able to seek out feedback and initiate this reflective process independently [6]. Being aware of one’s own thought processes and taking control of thinking and learning for development is known as metacognition [7]. These high-level capabilities may not be fully developed when students begin medical school [8].

Formative objective structured clinical exams (F-OSCEs) provide opportunities for students to be directly observed, assessed, and scored on their clinical skills by a standardized patient (SP). Feedback is typically scheduled after each station so students get immediate, face-to-face performance feedback from the SP before rotating to the next station.

Commercial simulation recording software provides the option to release more data to students after the event is over. Students can be given access to their scores, SP checklists, and video recordings of their performance. Ideally, students would spend time reviewing their performance in order to supplement and reinforce the SP verbal feedback [9]. In addition, as student self-review is not time-limited like SP feedback, students may discover areas for improvement, the SP missed or did not have time to discuss. Prior work on video self-review and performance reflection suggests a process can be created which aids in clinical skills development [10,11]. How often students take advantage of the opportunity to review their scores, checklists, and video recordings after a F-OSCE remains unclear. Prior medical education research suggests that students do not always fully use feedback that is available to them [12–15].

We set out to investigate how frequently students review F-OSCE performance data and whether this review improves performance on subsequent F-OSCEs. We also looked at the impact of student-faculty debriefing meetings on frequency of student review of data. Finally, we looked with a more longitudinal view to see if the frequency of student review impacts student performance on their summative OSCE (S-OSCE) at the end of the year.

## Methods

### Setting and participants

This study took place at the Frank H. Netter MD School of Medicine at Quinnipiac and was approved by the IRB. The importance of feedback and the role a student has in gathering and using feedback for performance improvement is explicitly introduced to students during medical school orientation, and frequently reinforced. Students are informed of the availability of F-OSCE performance data, trained in how to access it and encouraged to review and reflect on the scores, checklists, and video recordings. Standardized patients are trained to deliver effective verbal feedback and a quality control mechanism is in place that ensures ongoing monitoring of performance and continued education.

Medical students in their first and second year experience a series of F-OSCEs followed by an end of the year S-OSCE. First year medical students have five 3-station F-OSCEs over the course of nine months which is followed by an end of the year 5-station S-OSCE. Second year medical students have seven 2-station F-OSCEs and one 4-station F-OSCE over the course of nine months which is followed by an end of the year 5-station S-OSCE.

F-OSCE stations include 20 minutes with the patient followed by a 5-minute period where the SP completes a checklist and then 5-minutes of dedicated SP verbal feedback before the student rotates to the next station. SP’s score the students on history taking, physical exam, and communication. The students’ F-OSCE performance data is released for student self-review through the simulation recording software after the event. Performance data includes scores, checklists, and video recordings. Individual student-faculty debriefing meetings are scheduled for two F-OSCEs per year. These occur one week after their F-OSCE. Students are asked to prepare for these 20-minute meetings by reviewing their videos and annotating areas of strength and areas for improvement to discuss with the faculty member. The end of the year S-OSCEs mirror the F-OSCEs but do not include the immediate SP feedback. S-OSCE checklists, videos, and scores are not released in order to maintain exam security.

### Procedure

Simulation recording software automatically notes by date and time when a student accesses their scores, checklists and videos. This data is available to administrators. OSCE data from the 2015–2016 academic year was abstracted and de-identified. Student data was excluded if the student was not enrolled long enough to complete at least two consecutive F-OSCEs. The student OSCE scores and the metrics associated with student review were abstracted into an excel document for analysis.

### Analysis

Frequency of student review was analyzed using basic descriptive statistics. Data was analyzed both by medical school year and with all students combined. We first looked if frequency of review differed depending on whether a faculty debriefing was scheduled. This was tested by means of generalized estimating equations using a binomial distribution with a logit link and an exchangeable covariance. To assess the association of timely review of F-OSCE data and performance on a subsequent F-OSCE we used a linear mixed-model analysis. Fixed effects included the FOSCE number (i.e. time point), and indicator variables for whether a student viewed his/her score, checklist, or video. The intercept was random and varied by student with a identity structure. The timestamp of the data must have been before the subsequent F-OSCE to be considered timely and included in this part of the data analysis. Lastly, we used linear regression to see if the total number of score, checklist, and video reviews of F-OSCE data during the year predicted performance on the end of year S-OSCE. This analysis controlled for performance on the first F-OSCE. There was no time requirement for review of data for this analysis.

## Results

There were 92 first-year medical students, representing 460 opportunities for data review, available for analysis. There were 86 second-year medical students, representing 687 opportunities for data review, available for analysis. Two second-year medical students were excluded as they were not enrolled long enough to have sufficient data for analysis.

Both year 1 and year 2 students reviewed scores, checklists, and videos in decreasing frequency. Together year 1 and year 2 students reviewed 64% of the scores, 42% of the checklists, and 28% of the videos on their own. Frequency of review significantly improved when dedicated student-faculty debriefing meetings were scheduled (Table 1).

**Table 1.** Frequency of data review of F-OSCEs by years, with and without faculty debriefing meetings.

Combined	OSCEs with no faculty debriefing meeting N = 792	OSCEs with faculty debriefing meeting N = 355	z	P value	Odds ratio
Score	64%	88%	8.34	<.001	4.28
Checklist	42%	66%	6.99	<.001	2.69
Video	28%	92%	16.34	<.001	30.75
**Year 1 students**	OSCEs with no faculty debriefing meeting N = 276	OSCEs with faculty debriefing meeting N = 184	z	P value	Odds Ratio
Score	69%	88%	4.95	<.001	3.17
Checklist	51%	54%	0.96	.335	1.18
Video	46%	96%	8.32	<.001	26.19
**Year 2 students**	OSCEs with no faculty debriefing meeting N = 516	OSCEs with faculty debriefing meeting N = 171	z	P value	Odds ratio
Score	62%	89%	7.12	<.001	5.29
Checklist	37%	77%	9.39	<.001	5.86
Video	18%	88%	13.07	<.001	34.80

N = the number of individual student opportunities for review of OSCE data.

For year-1 students, the checklist review was associated with improved performance on the subsequent FOSCE (p = 0.038) by 1.07 percentage points on a scale of 0–100, but score and video review were not associated with improvement. For year-2 students, no review modality was significantly associated with improved performance on a subsequent F-OSCE (Table 2). For both groups of students, the total number of reviews during the year for each modality was not associated with improved S-OSCE performance (Table 3).

**Table 2.** Mixed-model results for modeling subsequent F-OSCE score.

	Regression coefficient	Std error	t	df	P value
**Year 1 students**					
Score	−0.13	0.66	−0.19	430.6	.849
Checklist	1.07	0.51	2.09	445.9	.038
Video	−0.34	0.59	−0.58	441.4	.562
Variances					
residual	16.92	1.26	ICC = .19		
intercept	4.04	1.14			
**Year 2 students**					
Score	−0.01	0.51	−0.03	644.4	.979
Checklist	0.66	0.49	1.33	628.0	.184
Video	0.36	0.52	0.69	662.3	.490
Variances					
residual	18.76	1.11	ICC = .20		
intercept	4.77	1.16

**Table 3.** Linear regression results for modeling end of year S-OSCE score.

	Regression coefficient	Std error	Beta	t	P value
**Year 1 students**					
Score sum	0.28	0.20	.186	1.41	.162
Checklist sum	−0.07	0.16	−.054	0.68	.681
Video sum	−0.26	0.21	−.150	−1.27	.206
Model summary					
R-square	0.32			F = 0.72	.580
MS error	4.05			df = 4,86	
**Year 2 Students**					
Score sum	−0.12	0.18	−.082	−0.65	.520
Checklist sum	0.26	0.15	.218	1.73	.087
Video sum	−0.03	0.24	−.013	−0.11	.910
Model summary					
R-square	0.14			F = 3.21	.017
MS error	10.93			df = 4,80	

## Discussion

This study provided some practical insights regarding medical student use of F-OSCE performance data. We expected the use of the data to vary by student but were surprised that checklists and video recordings were reviewed less than 50% of the time when students were left to their own accord. Our data probably overestimates student review in that we are only able to determine if a student accessed a video and cannot determine how much of the 20 minute videos they actually watched. Prior research also reports that student review of performance data is often limited and suggests a particular concern that the lower performing students are often those who do not review data [12,13].

One could speculate many potential reasons for lack of student data review. It is possible that students believe the five minutes of verbal feedback from the standardized patient is sufficient for skills development. Informal conversations with students suggest that emotions play a part in their behavior: some students don’t want to look at the data if they suspect it will make them feel incompetent (low score, missed several items on the checklist) or embarrassed (said something awkward in the video recording). First and second year medical students simply may not have not fully developed into self-directed learners able to engage in metacognition to improve performance. Regardless of the reason behind the lack of review, as medical educators we need to decide whether this data is valuable to students and, if so, put procedures in place to encourage or enforce review.

Our data demonstrates that faculty coaching in the form of individual student-faculty debriefing meetings are an effective strategy to ensure better use of available data. The disadvantage of meetings is the substantial use of faculty time. There are several alternative options to promote OSCE data review. Prior work demonstrated that a process of video review, informed self-assessment and reflection can improve OSCE performance without faculty involvement [10]. It might be worth adopting such a process and asking an administrator to track student review of data through the simulation recording software. Another option is peer assisted learning where students would be required to meet with a peer and review their data together [16]. Another consideration to improve data review is to make all OSCEs contribute to the overall course grade instead of only the end of the year S-OSCE. Perhaps weighting OSCEs in a manner analogous to quizzes and tests would encourage students to spend more time reviewing prior OSCE data to prepare for the next. A final consideration would be to assume students will not review OSCE data after the exam and schedule more time for SP feedback during the exam. However, it is unclear how much feedback a learner can absorb in real time [9]. A multi-modal approach that involves several of these strategies might be the best approach to reach more of these students.

Our research had another unexpected finding. We expected to see a more robust and consistent performance improvement on subsequent F-OSCEs for those students that reviewed the data. The only modality associated with improvement in performance was checklist review and that was only for year-1 students and amounted to 1.07 percentage points. While this is statistically significant it is unclear if this would be a clinically meaningful difference. Several factors may help to explain this outcome. The first factor to consider is that while each F-OSCE builds upon prior skills learned and practiced, we often test new skills or content. In particular, the physical exam and case content varies to coordinate with blocks of basic science course work (cardiology, pulmonology, etc). One may speculate that students who review their F-OSCE data are getting valuable feedback but subsequent F-OSCEs are not assessing the entirely same skill set. We also believe there may be unaccounted for variables in our research. Students may practice skills with each other or seek out coaching from faculty between F-OSCEs, which we could not account for in our research. There was also no difference in S-OSCE performance as a result of more data review. It should be noted that the scores for the S-OSCEs fall within a narrow range with most students achieving the expectations of the course, so a difference may exist but there is not enough variability in the scores to detect it.

Future work should explore the factors that impact student decision to review F-OSCE performance data. In-depth student interviews and qualitative analysis may provide some thematic answers to the questions raised in this study. Strategies to encourage data review could be directly compared to quantify impact. Finally, different endpoints for quantifying the value of performance data review beyond the year end S-OSCE exam should be considered. Performance on third-year clerkships would be one meaningful outcome for both faculty and students.

### Limitations

Beyond the limitations already mentioned, one should note that this is a single centered project. There may be cultural norms that drive student use of feedback data that are different at other institutions. However, despite the limitation in generalizability, we believe this information is valuable to understand how students interact with feedback data and how that can be augmented with an intervention.

## Conclusion

Medical students review F-OSCE checklists and videos less than 50% of the time when left to their own accord. Student-faculty debriefing meeting increases the use of this data by students. First year student review of checklist on F-OSCEs was associated with an increase in performance on subsequent F-OSCE, however this outcome was not observed with second year students.
